# Cow dung extract mediated green synthesis of zinc oxide nanoparticles for agricultural applications

**DOI:** 10.1038/s41598-022-22099-y

**Published:** 2022-11-27

**Authors:** Zoya Javed, Gyan Datta Tripathi, Mansi Mishra, Meghana Gattupalli, Kavya Dashora

**Affiliations:** Agri- Nanotechnology Laboratory, Centre for Rural Development and Technology, Indian Institute of Technology Delhi, New Delhi, 110016 India

**Keywords:** Biochemistry, Biotechnology, Environmental sciences, Nanoscience and technology

## Abstract

In the present study, zinc oxide nanoparticles (ZnO) were synthesized using cow dung extract to apply sustainable agriculture from rural resources. Studies on their antibacterial potential against *E. coli* DH* 5* alpha indicated lower antimicrobial activities than the bulk Zn and commercial Zn nanoparticles. Compared with control and commercial ZnO nanoparticles, the maximum seed germination, root length, and shoot length were observed after the priming of synthesized ZnO NPs. This study suggests that ZnO may significantly increase seed germination and have lower antimicrobial potential. Further, the lower in-vitro cellular leakage and reactive oxygen species (ROS) production provided new hope for using cow dung extract mediated nanoparticles for agricultural and industrial applications.

## Introduction

At present, nanotechnology has gained a lot of attention due to properties like the smaller size, higher strength, the high surface is volume ratio, excellent delivery, catalytic properties etc. Most importantly, it is observed that nanoparticles showed other characteristics than the bulk molecule of the same metals^1^. Due to the unique properties of nanoparticles, they have versatile applications in agriculture, food, and the pharmaceutical industry^2^.

The increasing of global population resulting in increased demand for food but reduced productivity due to climate change, soil fertility, and biotic and abiotic stress is a cause of great concern, and it plays a significant role in decreasing the crop yield. The world population in 2050 will be around 9.1 billion, i.e., approximately 34% higher than the existing population (FAO)^3^. Eventually, the requirement for food is probably increased as the same ratio^4^.

To conquer the issue, the utilization of manures, pesticides, disease and pest resistant varieties, and GMO crops have been increased significantly^5^. Chemical fertilizers are instrumental in improving the crop productivity, but their excess and unguarded use damaged the food quality and soil health^6–8^. Now a days, the applications of chemical and engineered nanoparticles (ENP) as fertilizers and for pest management have been increasing every year. To overcome the ecotoxicological impact of the chemical fertilizers and ENPs in soil system, researchers are trying to utilize biological and greener approach for the synthesis of nanoparticles. The biological extract from different plants and microbial sources utilized in the biosynthesis are safe, cost effective and having natural capping and reducing agents and phytochemicals like polyphenol, proteins and ascorbic acid^9^. Therefore, the scientists are exploring various plant extract, flowering, fruiting parts, microbial and cellular components, etc., for the green synthesis of the nanoparticles.

The ecofriendly and green synthesized nanoparticles may be significantly applied for various purposes like drug delivery, electrochemical and photo-degradation activities^10^, wastewater treatment, biofertilizers, antimicrobials and wound healing agents, pesticides and herbicides, packaging agents, nano-gel, etc. The phytochemicals in the extract not only play a role in the synthesis of the nanoparticles but have proven as non-toxic, anti-oxidative, and able to improve biocompatibility and stability also^11^.

Now a days, the zinc oxide nanoparticles are gaining a lot of attention due to the nontoxic behavior and higher requirement of the plants growth and development as well as the deficiency of Zn in soil^12^. Many researchers have used zinc oxide nanoparticles as a foliar fertilizer because they increase the agro morphological traits, photosynthesis, biomass, and yield of the plants^13^. Earlier, it was reported that ZnO nanoparticles show a significant effect on the germination and growth of wheat^14^. Zn^+2^ is an essential trace element for the metabolic process that occurs in plants as well as humans. The ZnO nanoparticles are considerably less toxic and declared as safe by FDA^9^.

Cow dung is one of the most abundantly available rural resources, which are pre-digested in cattle abdomen, and hosts various microorganisms facilitating soil conditioning, pest repellency and pathogen management. Conventionally, cow dung has been used extensively in organic and commercial agriculture. It can be used as a foliar application, soil drenching and as a seed treatment agent. By increasing resistance against biotic and abiotic stresses, enhanced output, improved shelf life, and improved quality of fruits, cow dung may act as key resources for producing healthy food from healthy soil in a healthy environment and non-toxic residues. Therefore, the present study focuses on the green synthesis of ZnO-NPs using cow dung extract. The biological synthesis and formation of ZnO-NPs have been optimized by varying the different factors like concentration, pH and temperature. Furthermore, different concentrations of the green synthesized ZnO-NPs were also employed to test the germination potential of Mung bean seeds at standard room temperature and pressure. As the continued decreasing of soil Zn level globally, our study provides a sustainable, ecologically safe and cost effective solution of the Zn deficiencies in soil for agriculture.

## Materials and methods

### Materials and reagents

The required chemicals from the study were purchased from SRL Chemicals Pvt. Ltd.; Nutrient medium was purchased from Himedia Pvt. Ltd. Nanoparticles were purchased from Sigma. Cow Dung was collected from the local gaushala (Cowshed). Mung beans were purchased from the local supermarket near IIT Delhi, India.

### Preparation of extract of cow dung

To prepare cow dung extract, 10 g of cow dung was added to 100 ml of distilled water. The mixture was then boiled for about 30 min and cooled and filtered using a muslin cloth. The filtrate was centrifuged at 10,000 rpm for 15 min. Pellet was discarded and supernatant was collected and filtered through Whatman filter paper no. 1. The filtrate was stored and further used for the synthesis of nanoparticles.

### Phyto-chemical analysis of the cow dung extract

The following methods were used to determine the photochemical and biomolecules present in the cow dung extract.

#### Total phenolic content

The total phenolic content of the extract was determined by using the method proposed by Luximon-Ramma et al.^15^ with slight modification. To determine the presence of total phenols in cow dung extract in terms of gallic acid equivalent per ml of extract, phenolic content was expressed.

#### Antioxidant activity

DPPH assay of the cow dung extract and nanoparticles was performed according to the method developed by Shimada et al. with some modification^16^. Ascorbic acid was used as the positive control, and DPPH with methanol was used as the negative control.

#### Total protein content

Total protein content in the extract was measured by the Bradford method and the protein was expressed in μg of BSA equivalent per ml of extract which has a maximum absorption at 595 nm^17^.

### GC–MS analysis of the extract

The ethyl extract was prepared by the previously described method with some modifications^18^ The sample was subjected to Shimadzu QP-2010 for the analysis loaded with NIST library. The average flow rate was approximately 1.21 ml/min. The other parameters applied as column oven temperature was 100 °C; injectable temperature 260 °C injection mode split; linear flow control mode; total flow 16.3 ml/min, plug flow 3 ml/min, and split ratio 10 for analysis^19^.

### Green synthesis of ZnO-NPs

To synthesize ZnO Nanoparticles, 90 ml of 0.02 M zinc nitrate hexahydrate solution was prepared and heated at 30 °C ± 2 °C for about 10 min. Cow dung extract was then slowly added while continuous stirring. The solution was then kept for 2 h to settle precipitate at the bottom. The solution was then centrifuged for 10 min. Pellet was collected and washed with deionized water. The formation of a creamy precipitate indicates the presence of zinc oxide nanoparticles (Fig. 2A) and resulting product was dried overnight at in hot air oven.

#### Effect of salt on the synthesis of zinc oxide nanoparticles

Zinc oxide nanoparticles were synthesized at zinc nitrate hexahydrate salt ranging from 0.01 to 0.04 M using cow dung extract and the peak was observed using UV–Vis spectrophotometer. It was observed that the zinc oxide nanoparticles were synthesized at a salt concentration of 0.02 M.

#### Effect of pH on the synthesis of zinc oxide nanoparticles

Zinc oxide nanoparticles were synthesized at pH ranging from 5 to 8 using cow dung extract, and the peak was observed using a UV–Vis spectrophotometer. It was observed that the zinc oxide nanoparticles were synthesized at pH 8.

#### Effect of temperature on the synthesis of zinc oxide nanoparticles

Zinc oxide nanoparticles were synthesized at temperatures ranging from 30 to 70 °C using cow dung extract and the peak was observed using a UV–Vis spectrophotometer. It was observed that the zinc oxide nanoparticles were synthesized at 70 °C.

### Characterization of synthesized nanoparticles

The formation of ZnO nanoparticles was primarily characterized by Ultraviolet–Visible (UV–Vis) spectrophotometer (Shimadzu, double beam UV–Vis Spectrophotometer). The solution absorption maxima at wavelengths between 200 and 800 nm were scanned primarily to describe the synthesis of ZnO. X’Pert PRO diffractometer (PANalytical Netherlands) was used to characterize the synthesized ZnO performed the X-ray diffraction. It was run from 20° to 80° at 0.041°/minute with a time constant of 2 s. Scanning electron microscopy (SEM) (Zeiss EVO Series EVO 18) and Energy dispersive spectroscopy (EDX) was used to confirm the elemental composition and morphology of the synthesized ZnO. Using a Thermo Nicolet-IS-50, Fourier-transform infrared (FTIR) spectroscopy in the 4000 to 400 cm^−1^ range was carried out. The ZnO spectra were acquired using KBr pellets.

### Antibacterial potential of biosynthesized zinc oxide nanoparticles

#### Disc diffusion method

The biosynthesized ZnO-NPs were tested for their antibacterial vigor against *E. coli* DH 5 Alpha by the disc diffusion method. About 500 µl (1.5 × 10^8^ CFU/ml) of a fresh *E. coli* cultures had been spread on nutrient agar medium. After spreading, the sterile discs preloaded with 10 mg/ml concentrations of biological ZnO, commercial ZnO and bulk zinc nitrate are transferred on plates under aseptic conditions and incubated at 30 °C. After 24 h, the clear zone that formed around the discs was measured (Fig. 5B) and tabulated^20^.

#### Minimum inhibitory concentration (MIC)

The broth micro dilution method was used to evaluate the MIC of biosynthesized ZnO-NPs. As a stock solution, 100 mg/ml of biosynthesized ZnO-NPs was used. Each 96 wells in the ELISA plate had around 100 µl of NB added to it before an equivalent volume of serially diluted ZnO-NPs stock solution was added. 10 µl of *E. coli* DH 5 Alpha suspension (1.5 × 10^8^ CFU/ml) was added to each well, and the plates were then incubated in an incubator for 24 h at 30 °C. After 24 h incubation, add 20 µl of Tetrazolium chloride (2 mg/ml) and incubated for 30 min in the dark. The MIC was determined to be the observed color change in the wells.

### Effects of the green synthesized ZnO NPs on protein leakage and sugar leakage

The Green synthesized ZnO nanoparticles from cow dung extract were tested for protein leakage against *E. coli* DH 5 Alpha was performed according to the study of Ghabban et al.^21^. In concise, the *E. coli* DH 5 Alpha was treated with 1 mg/ml ZnO NPs was incubated at 30 °C with shaking for 24 h. After the incubation, 1 ml of cell suspension was centrifuged at 5000 rpm for 1 min; after that, 0.5 ml of the suspension is supernatant was mixed with 2.5 ml of Bradford reagent. Finally, the amount of protein was estimated with the help of Bradford’s method (1976) using a UV–Vis spectrophotometer^17^.

The effects of ZnO NPs on metabolic reactions of bacterial cells may be calculated by cytoplasmic leakage. Nanoparticles cause the bursting of bacterial cells, which releases their intracellular substances like proteins and sugars. Dinitrosalicylic acid method^22^ was used to determine the leakage of reducing sugars from bacterial strains after treating them with 1 mg/ml concentrations of ZnO NPs. The treated *E. coli* DH 5 Alpha cells were incubated for 24 h at 30 °C. After incubation, the bacterial suspension was centrifuged at 4000 rpm at 4 °C for 1 min. Following this, 0.5 ml of supernatant was taken and mixed with 3 ml of DNS (3,5-dintrosalicycline acid) reagent. Tubes were then heated at 90 °C for 10 min. After cooling, OD was measured at 540 nm to estimate the leakage of reducing sugars from test samples. Experiments were performed in triplicates^23^.

### Determination of the reactive oxygen species (ROS) production

The reactive oxygen species was determined by the method previously described by Chaudhary et al.^24^. Overnight treated cells with nanoparticles washed and 10 mM DCFH-DA was applied for 30 min. After washing with PBS, the cells were resuspended and analysis in CLSM at 488 nm excitation and 552 nm emission.

### Impact on seed germination

The seed germination test was carried out by the floatation method^25^. Mung bean is a  frequently consumed legume in India and is widely used for germination studies. The OECD (Organization for Economic Cooperation and Development, 1984) the study of phytotoxicity has already recommended the plant. The plant studies comply with the relevant Institutional, National, and International guidelines and legislation. The species are ranked as least concerned under the IUCN guidelines. The Mung beans (*Vigna radiata*) were obtained from the local market superstore in New Delhi, India, a beaker of 50 ml water, and allow standing for 10 to 15 min. About 70 seeds were surface sterilized with 0.1% Sodium hypochlorite and washed thoroughly with distilled water 2 to 3 times. Then seeds were soaked in different concentrations of ZnO nanoparticles (5%, 10%, 25%, 50%, 75%, and 100%) and control for two hours in the shaker at 30 °C temperatures in 50 ml of deionized solution. After 2 h, the seeds were transferred in Petri plates containing moistened filter paper. The Petri plates were then placed at room temperature for 5 days. After incubation for 5 days, the germination percentage, root length and shoot length were calculated for all samples^26^.

### Statistical analysis

All the experiments were performed in triplicates and data were analyzed with the help of MS-Excel.

## Result and discussion

### GC MS of cow dung extract

The GC-MS data represent availability of various organic compounds present in the extract. There are 33 available photochemical peaks observed in the sample. It is considered that the presence of photochemical are mainly responsible for synthesizing metallic nanoparticles^27^. The major compound presence in the extract are tetraethylene glycol (17.57%), 2-Ethoxyethyl methyl phthalate (17.40%), Palmitic Acid (16.21%), Myristic acid (5.57%), 2-Propanol, 1-chloro-, phosphate (5.41%), Oleic acid (5.13%),Phthalic acid, 3-chlorophenyl methyl ester (4.52%), Hexadecanoic acid (2.44%), Stearic acid (2.26%). Most of these compounds are reported as useful component in nanoparticle synthesis and perform various functions like stabilizers and coating agents in nanoparticle synthesis^28–32^. A detailed list of the compounds and chromatogram are attached with Supplementary Information.

### Characterization of synthesized zinc oxide nanoparticles

**Figure 1:**
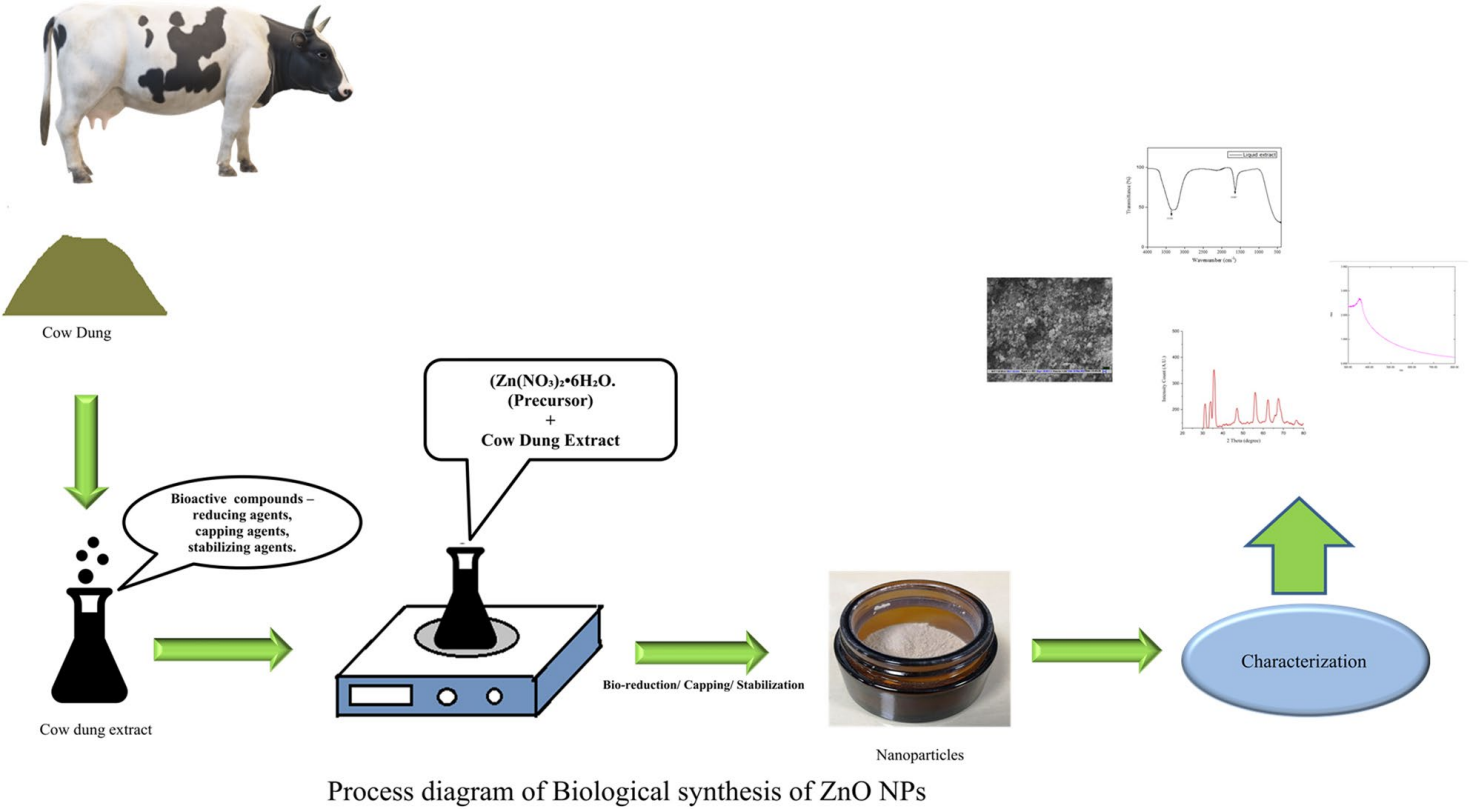
Synthesis of ZnO nanoparticles from cow dung extract.

The biological synthesis of nanoparticles is a novel approach and eco-friendly process. Synthesis of nanoparticles from plant extract and microbes are reported in various literatures^33–36^. The presence of various types of biomolecules (phytochemicals and biochemicals) in plant extract and microbial extracts (polyphenols, alkaloids flavonoids, antioxidants, proteins, enzymes etc.) are mainly responsible for the reduction, capping and stabilization of the nanopaticles^37,38^. It has been extensively reported that cow dung is a potential source of various microbial enzymes antimicrobial substances^39^. The synthesis of nanoparticles from the cow dung extract is an affordable and narrative process. The phytochemical analysis of the extract is carried out to detect the critical constituent in the extract is present in Table 1. As described in the previous section, it is observed from GC–MS that cow dung extract having variety of the bioactive compounds and some of them are reported as the potential stabilizers and reducers for the synthesis of various metallic nanoparticles. The flow diagram of the synthesis process of ZnO nanoparticles is given in Fig. 1. Although the exact mechanisms of the biosynthesis are not well understood. Still, it is reported that various molecules such as amines, phenol, enzymes, and other proteins in the biological extract have a reducing effect on metal salts^40^, possibly responsible for the biosynthesis of the metal oxide nanoparticles.

**Table 1:** Phytochemical screening of cow dung extract.

Name of the test	Results
Total phenol	0.12 µg/ml
Antioxidant	5.6%
Total protein	2.24 µg/ml
Antioxidant nano	4.7%

Sutradhar and Saha, used tomato extract to synthesize zinc oxide nanoparticles^41^. The presence of phytochemicals in the tomato functioned as a natural capping and reducing agent. Phenols and protein in tomato plays a crucial role in reducing zinc oxide, and flavonoids and flavones present in the extract act as the capping and reducing agent for synthesizing zinc oxide nanoparticle^42^.

**Figure 2:**
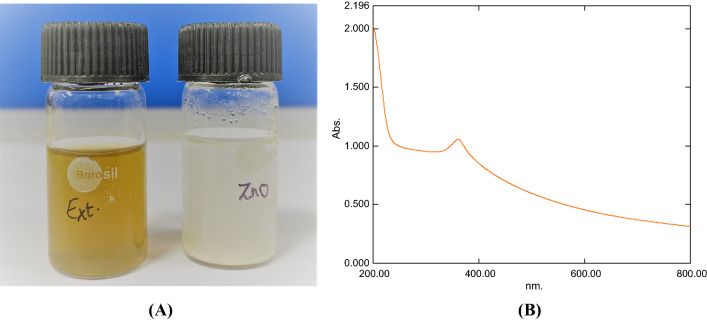
(**A**) Cow dung extract and synthesized nanoparticles. (**B**) UV–Vis spectra of the nanoparticles.

In this study, after synthesis, the white precipitate of ZnO was dried in hot air oven to convert into zinc oxide nanopowder. When the powder was redissolved in deionized water, UV–Vis spectra were observed, and the absorption peak at 360 nm (Fig. 2B). The sharpest peak of all the differences was seen in the Nano powder generated under optimized conditions, which was then dried and stored for further investigation at room temperature. To maximize the synthesis, higher doses of zinc nitrate hexahydrate were utilized. When zinc nitrate hexahydrate concentration was increased from 0.02 to 0.04 M, an increase in absorption and a sharpness of peak were seen (see Supplementary DataSupplementary Data attached). However, as the concentration was raised to 0.02 M, the absorbance dropped and the peak’s width significantly widened. Thus, it was determined that decreasing the synthesis of nanoparticles was caused by increasing the concentration of metal ions above a threshold value.

The pH and temperature of the reaction mixture are two additional important variables in the green synthesis of nanoparticles. The pH is one of the crucial factors that may affect the reduction of metallic salt. It might be possible that changes in the pH may affect the reducing and capping of the phytochemicals, which ultimately influence the growth of the nanoparticles^43^. In this study, it was shown that increasing the pH from 8 to 12 increased the absorbance of the final result, and that the pH 10 spectrum showed a characteristic absorption peak with an almost straight absorption line (see Supplementary Data attached). However, pH 8 was found to be better for both absorbance and sharpness. It is reported that a higher level of OH^**−**^ influences the formation of Zn–O bond due to increased attraction of Zn^+^ at higher pH^44^. According to Mohammadi and Ghasemi^43^, the optimum pH for the synthesis of ZnO nanoparticles is a subset of the substances which used as stabilizing and capping agents. Similar results occur in the study done by Nagarajan and Kuppusamy^45^, who found a characteristic absorption peak at pH 8.

Similarly, it is well known that one of the critical variables in the creation of nanoparticles is temperature. Similar patterns with low and incredibly broad peaks could be seen in the spectra at 70 °C. At 80 °C, the absorbance increased significantly, but no obvious peak was seen. The optimization reactions did not result in any significant peak shifts. It is observed that higher temperature increases the rate of reaction, which helps in the reduction of size of the nano range. As the increasement of the temperature occurs, there is sharpen of the peaks (narrow SPR) were found^46^. Rahayu et al. synthesized ZnO nanoparticles by using fruit extract of *Averrhoa bilimbi* as reducing and capping agents. They observed that during the synthesis process at 70 °C^47^. The formation of deep white precipitate and faster evaporation of solvent has occurred.

### FTIR spectroscopic analysis

**Figure 3:**
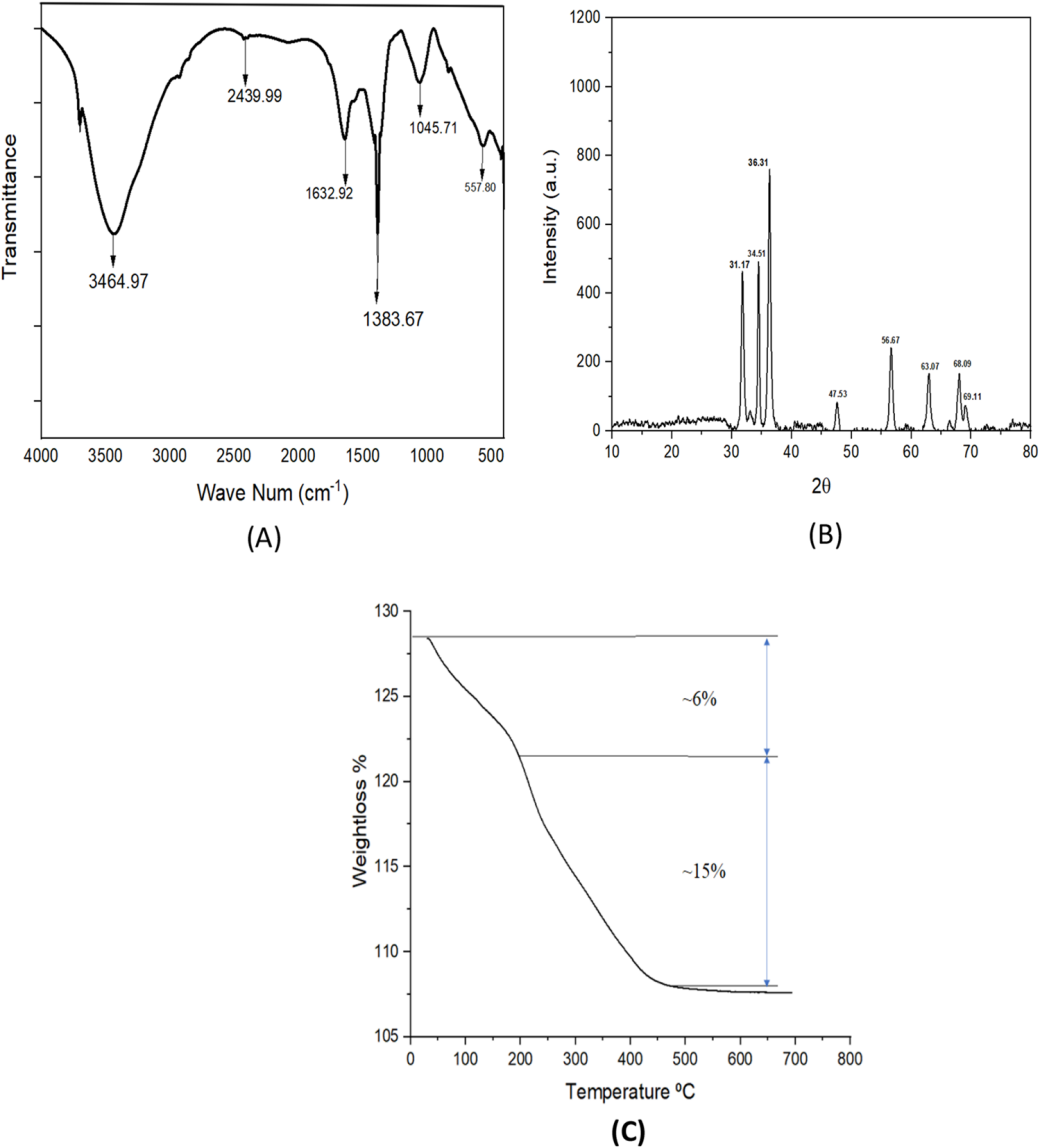
(**A**) FTIR spectra of ZnONps. (**B**) XRD analysis of the synthesized ZnONps. (**C**) Thermogravimetric analysis of synthesized ZnO Nps.

Fourier Transform Infrared spectroscopic analysis confirmed the presence of various functional groups, which may involve the synthesis of ZnO nanoparticles. The techniques indicate the possible presence of phytochemicals, which may be responsible for the capping and reduction process during the green synthesis ZnO nanoparticles. Figure 3A representing the FTIR spectra of synthesized by the green approach showed a peak at 557.80 cm^−1^ which is corresponding to the hexagonal ZnO symmetric bending vibration. The broad peak at 3464.94 cm^−1^ and 1045.71 cm^−1^ signifies the OH and C–OH stretching vibrations respectively. Some other peaks 2439.99, 1632.92 as well as 1383.67.39 cm^−1^ show the presence of different groups (with, C=C, C–H C–O stretching). Our studies align with the findings of Jayarambabu et al., with almost similar peak positions^48,49^.

### XRD analysis

The crystalline structure of the nanoparticles was confirmed with X-ray diffraction studies and the diffraction pattern of the cow dung synthesized ZnO Nps can be observed in Fig. 3B. The crystalline peaks were positioned at (2θ) peaks angles of 31.80°, 34.45°, 36.28°, 47.59°, 56.65°, 62.94°, 66.46°, 68.00°, 69.09°, 72.57° and 77.06° which is correlated with planes (100), (002), (101), (102), (110), (103), (200), (112), (201), (004), (202) respectively. All the above angles and planes indicate the characteristics hexagonal structure of ZnO nanoparticles corresponding to the JCPDS Card No. 04-007-9804. Studies done by Gupta et al. showed very similar results of the green synthesized ZnO nanoparticles indicating good agreement with our results^50^. Recently, Shubha et al. also synthesized ZnO nanoparticles by the help of Cow dung and found a very similar peaks and hexagonal structures^51^.

### Thermo-gravimetric analysis (TGA) of ZnO NPs

TGA assists in finding out the thermal stability of the nanoparticles and helps identify the volatile component content via measurement of weight loss at a constant rate of heat^52^. The figure represents ~ 6% weight loss in the initial stage due to the water loss (Fig. 3C). The weight loss up to 200 °C is due to water loss. i.e., the loss of physically and chemically adsorbed water molecules to the ZnO NPs^53,54^. As the losses increase, the surface hydrophobic of ZnO NPs also enhances and results in the loss of water molecules. As shown in the Fig. 3C, weight loss of ~ 15% at the second stage is due to the decomposition of the capping agents from the cow dung extract that are adsorbed over ZnO NPs. Li et al. also showed that the weight loss after 250 °C might be due to the reduction of oxygen-containing functional groups on the surface of the nanoparticles^55,56^. This is also due to CO_2_ removal, as the peak near 250 °C represents an endothermic reaction. After 450 °C, no further degradation occur, representing the completion of the evaporation of water molecules and decomposition of organic components over ZnO NPs^57^. The retained percentage after 450 °C represents the presence of ZnO NPs along with some inorganic material. This shows that ZnO NPs synthesized from cow dung extract are bound by various functional groups as capping agents and act as green nanoparticles.

### SEM, EDAX and AFM analysis

**Figure 4:**
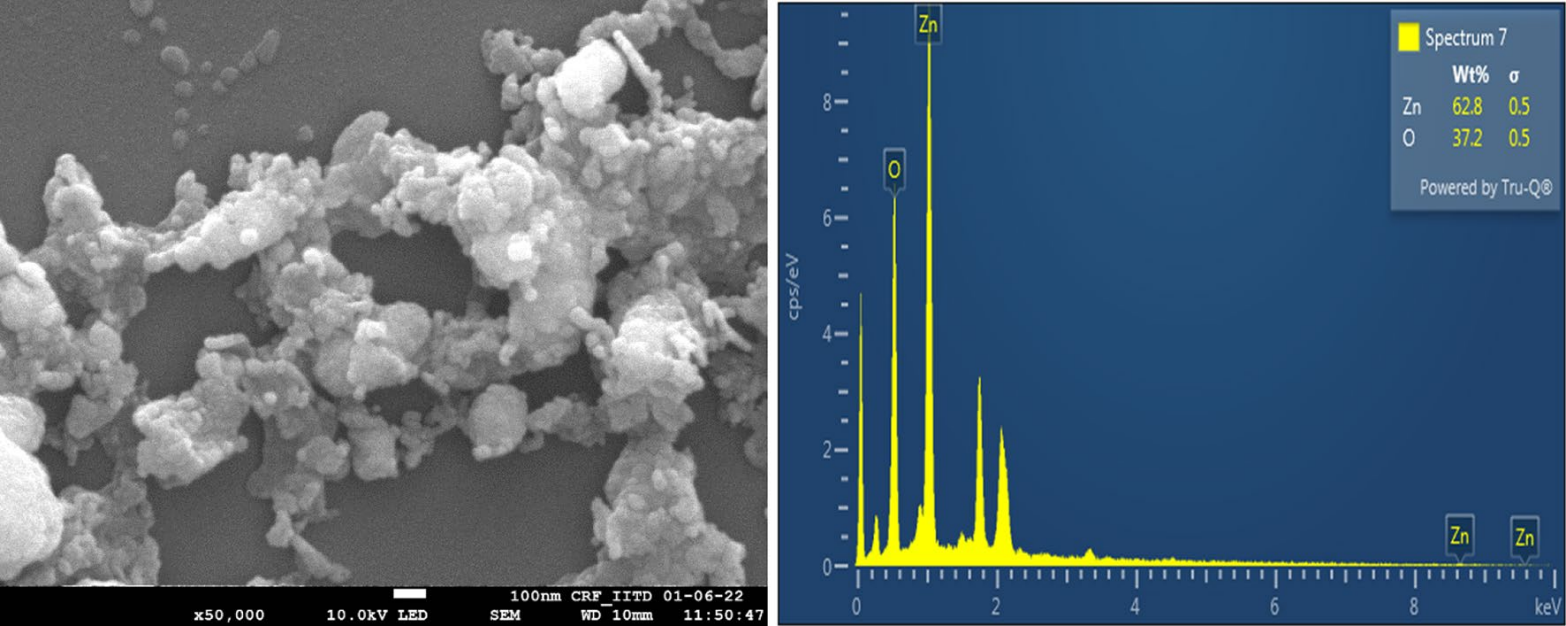
SEM and EDX of cow dung extract mediated biologically synthesized nanoparticles.

**Figure 5:**
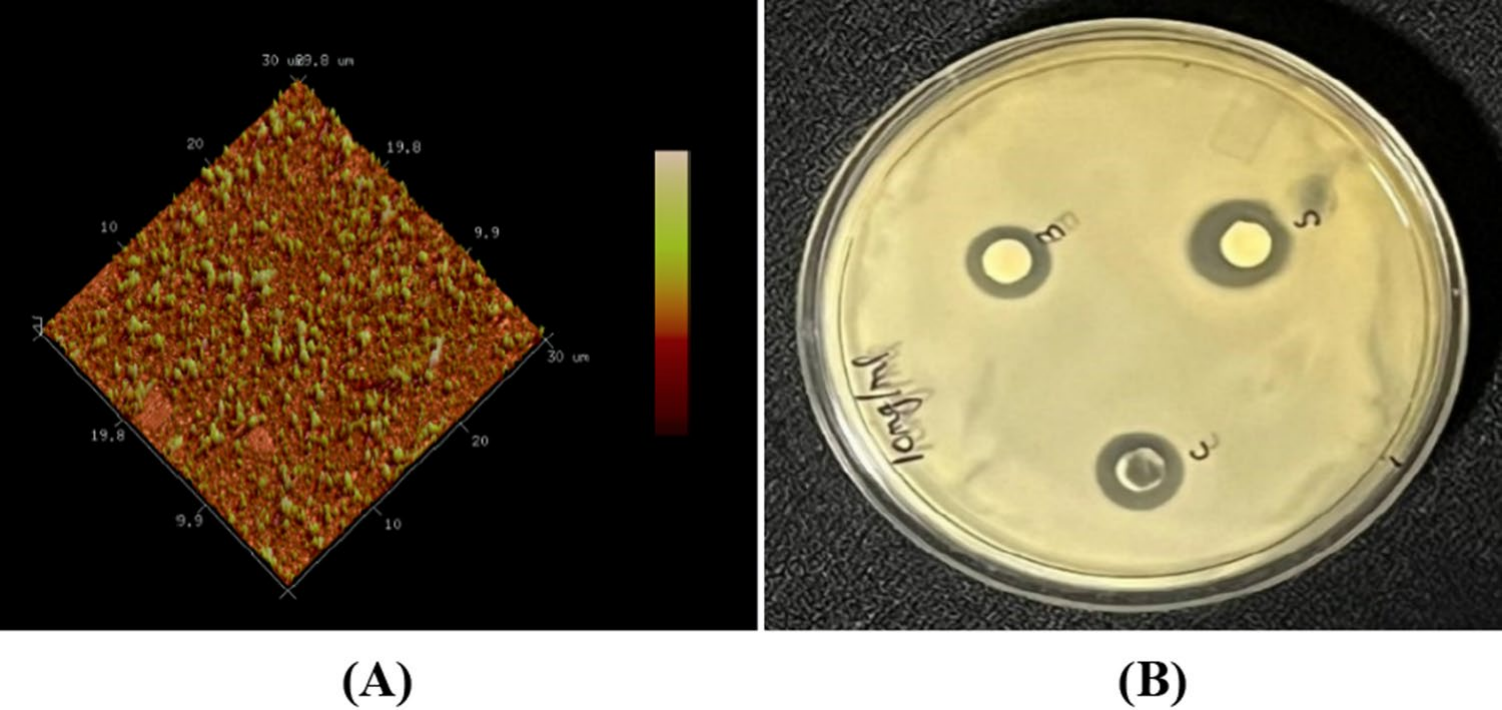
(**A**) AFM image of ZnO nanoparticles. (**B**) Disc difussion test against *E. coli.*

The presence of Zn and O synthesized ZnO was confirmed by EDAX spectrum. The synthesized nanoparticles were under nanoscale (1–100 nm range) and were arranged in a spherical and hexagonal form (Fig. 4). The topography of the synthesized nanoparticles was shown with the help of Atomic Force Microscopy (Fig. 5A). In previous studies, Suganya et al. synthesized ZnO nanorod using vermiwash extract prepared from cow dung and leaf litter, found average size and width in the range of 65 and 213 nm^58^.

### ZnO nanoparticle priming on seed germination

Zn is one of the essential micronutrients and helpful in germinating seeds, developing plant and crop yields, and providing resistance to various biotic and abiotic stresses^12^. Although the deficiencies of the Zn is increasing in soil worldwide, the over application is also a serious threat to soil health and plants^59^. Therefore, the researchers have proposed the application of ZnO nanoparticles across the world. A diverse range of impacts of the ZnO nanoparticles have been reported to date, still, there is a significant gap of the studies of ZnO nanoparticles on seed germination and plant growth^60^. Recently, it was observed in the various studies that seed priming with biologically synthesized ZnO nanoparticles positively affected in seedling and other related metabolic activities in several common staple crops like rice, maize, and wheat etc^12,23,61,62^.

**Figure 6:**
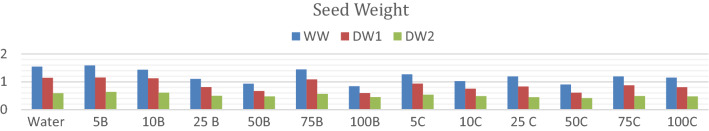
Effect of different concentration of ZnO nanoparticles on the seed germination weight (*WW* wet weight, *DW1* dry weight day 1, *DW2* dry weight day 2).

**Figure 7:**
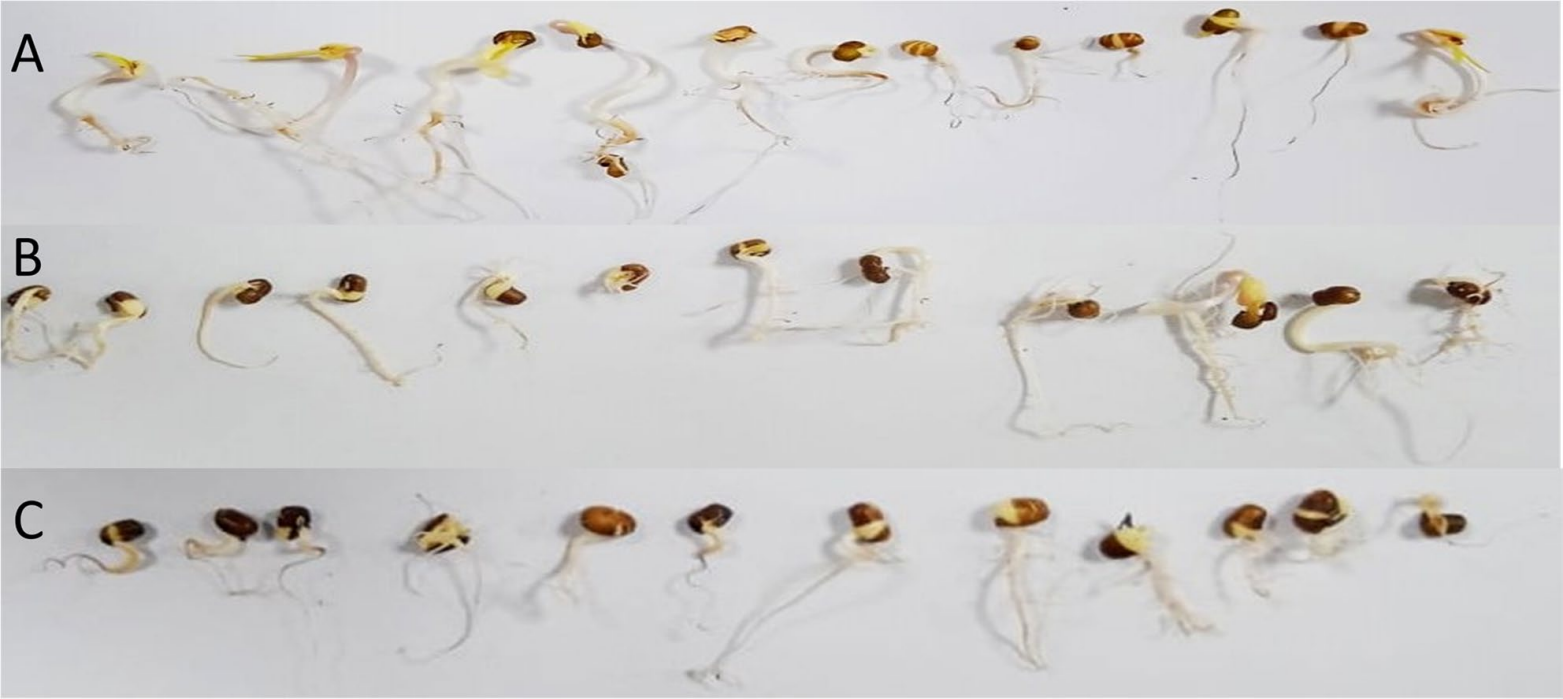
Germination of mung bean seeds (**A**) control, (**B**) seedes primed with biological ZnO nanoparticle. (**C**) Seeds primed with ZnO chemical nanoparticles.

To investigate the role of ZnO nanoparticles on seed germination, seedling parameter and dry matter production (DMP) were investigated under laboratory conditions (Figs. 6, 7) by priming the Mung bean seeds with different concentrations of ZnO nanoparticles. It was observed that the seeds primed with 5 mg/ml concentration of biological ZnO nanoparticles showed higher wet and dry weight compared to different priming concentrations. In the past, ZnO nanoparticles (1500 ppm) primed maize seeds indicated a higher vigor index in compare with the control^12^. Table 2 presents the length of seedlings after 4 days of incubation for the seeds primed with 10 mg/ml of nanoparticles. It is observed that the biologically synthesized nanoparticles are more effectively induced in the seedling than the chemical nanoparticles^63,64^.

**Table 2:** Effect nano priming on seedling of the mung bean after 4 days.

Name of the sample	Average shoot length (mm)	Average root length (mm)	Total seedling (mm)
Control	35.2	52.2	87.4
Biological Nps	39	23.8	62.8
Chemical Nps	27.8	31	58.8

In past several studies found ZnO nanoparticles exposure increased the root and shoot elongation in wheat, sweet sorghum, and soybean. Recently, compared to the traditional hydro-priming method, rice seed nano-primed with biological ZnO nanoparticles at low concentration (10 mg/l) considerably improved 100% seed germination and seedling metrics^65^. The precise process by which nanoparticles promote seed priming is not well defined in the literature.

A few studies have suggested theories and experiments in which nanoparticles are introduced into seed coat pores to boost water molecule penetration and stimulate the activity of enzymes that break down starch and produce ROS, hence physiologically enhancing seed germination^35,66^. Seed firms may pursue nano-material-based priming agents as a substitute method for traditional and commercial seed priming. The current work aims to develop organically synthesized nano-material based on cow dung for future good agricultural practices and seed priming applications in the agri-seed business.

### Antimicrobial activity of synthesized ZnO nanoparticles

The microbial ecotoxicity of ZnO nanoparticles is a severe concern for field application. From an ecological perspective, few studies are available, and most of the studies are focused on the impact of nanoparticles on pathogenic microorganisms^67^. Microbial species like *Bacillus subtilis, Pseudomonas* etc., used for the ecotoxicological studies^68^. The ZnO nanoparticles interact with the microorganisms’ outer membrane of the microbial cell wall, resulting in the breakdown of the plasma membrane, and thus permeability may also change. So, nanoparticles easily cross the membrane and entered into the cytoplasm and causing a negative impact on cellular activities like the growth of the cells^69,70^.

ZnO NPs also generate various other reactive oxygen species, such as hydroxyl radicals and singlet oxygen, stimulating cell death^71^. In presently available studies, the ZnO NPs produced from green synthesis processes have shown significant antifungal and antibacterial properties. Table 3 lists MIC and Zone of inhibition values observed for test microbe *E. coli* were found to be more sensitive for chemical nanoparticles. The MIC value for our synthesized nanoparticles is 1.6 mg/ml. Thus, nanoparticles can be used safely as plant nutrients. Nanotoxicity is a significant concern that influences the application of ZnO nanoparticles as a biofertilizer. For the safe application of ZnO nanoparticles, it is required to analyze nanoparticles’ toxicity on microbes. Our results proposed minimum toxicity compared to the bulk zinc nitrate hexahydrates and the commercial Zn nanoparticles. These preliminary results provide hope for the suitable soil application of our synthesized ZnO nanoparticles. For a better understanding, the reactive oxygen species studies are also done in the next section.

**Table 3:** Comparative analysis of the synthesized nanoparticles and chemical nanoparticles.

Name of the test	ZnO biological	ZnO chemical
Zone of inhibition	3.3 mm	3.75 mm
MIC	1.6 mg/ml	0.78 mg/ml
Protein leakage	0.8 µg/ml	0.7 µg/ml
Sugar leakage	4.56 µg/ml	5.92 µg/ml

### Effect of ZnO nanoparticles on protein and sugar leakage and ROS production

**Figure 8:**
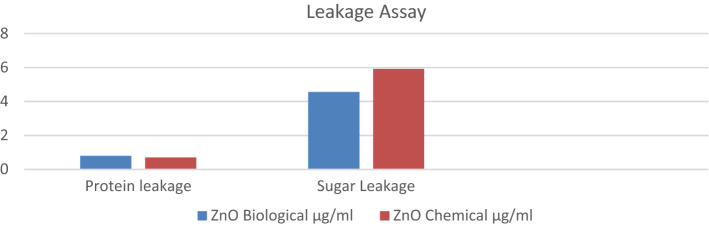
Comparative protein and sugar leakage assay under treatment of ZnO nanoparticles of *E. coli*.

The cytoplasmic leakage of various molecules like protein and sugars are considered as the marker for membrane permeability and damage. Yusuf et al. reported that the molecules’ cellular leakage may be due to the bactericidal activity of ZnO Nanoparticles (Fig. 8) comprises the cellular leakage of the microorganisms *E. coli*^72^. The results obtained in the present experiment indicated higher sugar leakage in chemical nanoparticles (Table 3), so biological nanoparticles from cow dung extract are comparatively safe.

**Figure 9:**
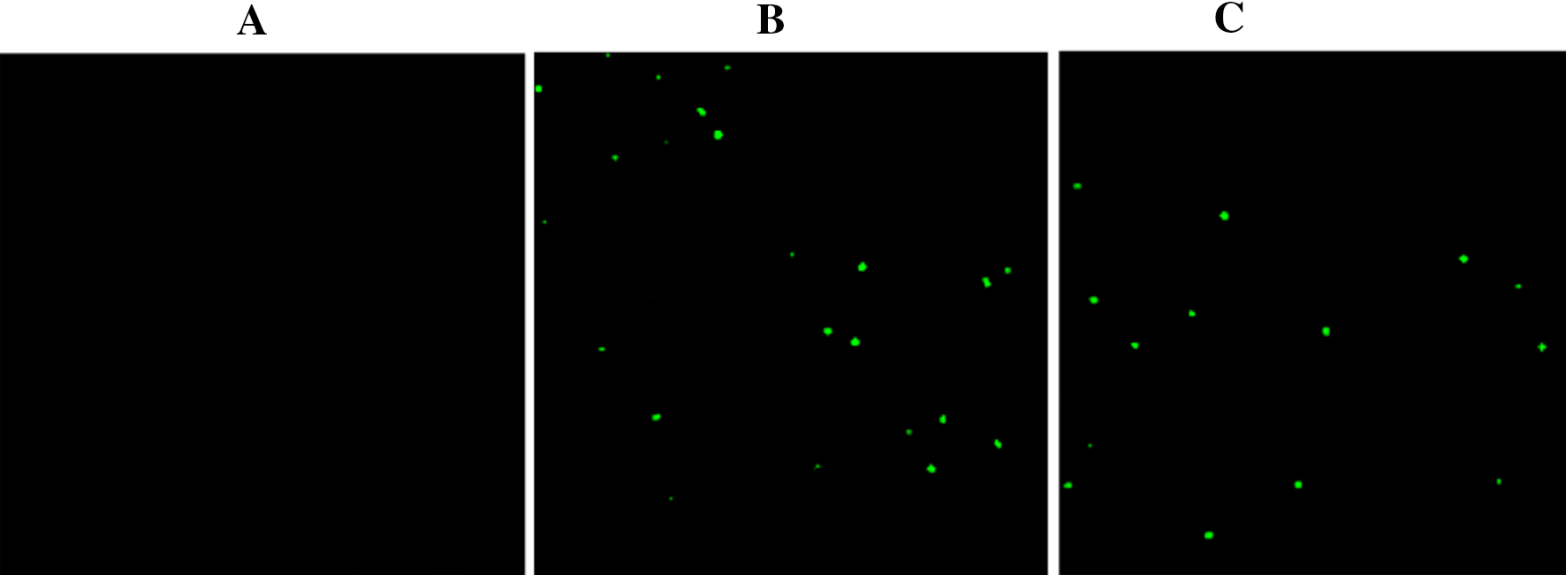
DCF-DA mediated ROS production in *E. coli* (**A**) control, (**B**) *E. coli* treated chemical Nps. (**C**) *E. coli *treated biological synthesized Nps.

The reactive oxygen species (ROS) under stressed conditions is a vital stress indicator of the different living microbes. The excessive production of ROS may cause cell death. Yusuf et al. explained the detailed chain of reactions, which ultimately cause microorganism cellular death^72^. They said that ZnO nanoparticles may produce ROS in the microorganisms *E. coli.* In our study, the production of ROS was determined qualitatively with the help of CLSM. Figure 9 indicates that biologically synthesized ZnO nanoparticles have produced lower ROS in the *E. coli* cells than the chemical ZnO nanoparticles.

## Conclusion

The study ‘green synthesis of zinc oxide nanoparticles using cow dung extract’ presenting a novel approach of eco-friendly and cost-effective process of ZnO nanoparticles synthesis from a rural resource i.e. cow dung. The present study is the first step toward a sustainable solution of soil Zn deficiency and seed priming with the help of ZnO nanoparticles originating from cow dung, utilized in agriculture since ancient times. In the comperison of the commercial ZnO nanoparticles, the cow dung mediated synthesized ZnO particles are safe because of the lower toxicity on microbes. So, the present studies showed the constructive outcome of ZnO NPs seed germination and Nano-priming could be an effective technique for the enhanced seed germination of crops Therefore, the opportunity to use nutrients such as Zn at the nanoscale level might be a vital step in agriculture. Overall, the outcomes of the study will be helpful to the nano fertilizer industries to choose nano-fertilizer production, particularly ZnO NPs that could be utilized as supplement source to decrease the Zn deficiency in plants. However, there should be an extensive study on plant growth as well as the ecotoxicological impact on the soil microbial community is still required. The present study represents only the preliminary results on seed germination and selected microorganism. Whereas, various parameters of seed germination studies, toxicity on different soil types in rural setups, and even the synthesis process requires a more detailed study for the development of cow dung mediated ZnO and other agriculturally important nanoparticles for sustainable agriculture.

## Supplementary Information

Supplementary Information.

## Data Availability

Data produced in the study are included in the paper and Supplementary Files. Additional data will be provided as per the requirements by the corresponding author (Prof. Kavya Dashora).

## References

[1] Ahmad T, Ganguly A, Ahmed J, Ganguli AK, Alhartomy OAA (2011). Nanorods of transition metal oxalates: A versatile route to the oxide nanoparticles. Arab. J. Chem..

[2] Naveed Ul Haq A (2017). Synthesis approaches of zinc oxide nanoparticles: The dilemma of ecotoxicity. J. Nanomater..

[3] FAO (2009). Executive Summary Proceedings of the Expert Meeting on How to Feed the World in 2050.

[4] Kumar KG, Kumaravelu N, Sivakumar T, Gajendran K (2006). Study on Panchakavya—An indigenous formulation and its effect on the growth promotion of crossbred pigs. Indian J. Anim. Res..

[5] Yadav S, Subhash B, Yadav MK, Singh K, Yadav GS, Pal S (2013). A review of organic farming for sustainable agriculture in Northern India. Int. J. Agron..

[6] Zamir D (2001). Improving plant breeding with exotic genetic libraries. Nat. Rev. Genet..

[7] Conley DJ (2009). Hypoxia-related processes in the Baltic Sea. Environ. Sci. Technol..

[8] Bai Y-C (2020). Soil chemical and microbiological properties are changed by long-term chemical fertilizers that limit ecosystem functioning. Microorganisms.

[9] Senthamarai MD, Malaikozhundan B (2022). Synergistic action of zinc oxide nanoparticle using the unripe fruit extract of *Aegle marmelos* (L.)—Antibacterial, antibiofilm, radical scavenging and ecotoxicological effects. Mater. Today Commun..

[10] Alshehri SM (2018). Synthesis, characterization, multifunctional electrochemical (OGR/ORR/SCs) and photodegradable activities of ZnWO4 nanobricks. J. Sol-Gel. Sci. Technol..

[11] Alshameri AW, Owais M (2022). Antibacterial and cytotoxic potency of the plant-mediated synthesis of metallic nanoparticles Ag NPs and ZnO NPs: A review. OpenNano.

[12] Itroutwar PD, Kasivelu G, Raguraman V, Malaichamy K, Sevathapandian SK (2020). Effects of biogenic zinc oxide nanoparticles on seed germination and seedling vigor of maize (*Zea mays*). Biocatal. Agric. Biotechnol..

[13] Munir T (2018). Effect of zinc oxide nanoparticles on the growth and Zn uptake in wheat (*Triticum aestivum* L.) by seed priming method. Digest J. Nanomater. Biostruct..

[14] Du J (2017). Can visible light impact litter decomposition under pollution of ZnO nanoparticles?. Chemosphere.

[15] Luximon-Ramma A, Bahorun T, Soobrattee MA, Aruoma OI (2002). Antioxidant activities of phenolic, proanthocyanidin, and flavonoid components in extracts of Cassia fistula. J. Agric. Food Chem..

[16] Shimada K, Fujikawa K, Yahara K, Nakamura T (1992). Antioxidative properties of xanthan on the autoxidation of soybean oil in cyclodextrin emulsion. J. Agric. Food Chem..

[17] Bradford MM (1976). A rapid and sensitive method for the quantitation of microgram quantities of protein utilizing the principle of protein-dye binding. Anal. Biochem..

[18] Zaman NR (2021). Plant growth promoting endophyte Burkholderia contaminans NZ antagonizes phytopathogen *Macrophomina phaseolina* through melanin synthesis and pyrrolnitrin inhibition. PLoS ONE.

[19] Singh H (2021). Recent advances in the applications of nano-agrochemicals for sustainable agricultural development. Environ. Sci. Process. Impacts.

[20] Umavathi S (2021). Green synthesis of ZnO nanoparticles for antimicrobial and vegetative growth applications: A novel approach for advancing efficient high quality health care to human wellbeing. Saudi J. Biol. Sci..

[21] Ghabban H (2022). Antibacterial, cytotoxic, and cellular mechanisms of green synthesized silver nanoparticles against some cariogenic bacteria (*Streptococcus mutans* and *Actinomyces viscosus*). J. Nanomater..

[22] Miller GL (1959). Use of dinitrosalicylic acid reagent for determination of reducing sugar. Anal. Chem..

[23] Sharma P, Goyal D, Chudasama B (2022). Antibacterial activity of colloidal copper nanoparticles against Gram-negative (*Escherichia coli* and *Proteus vulgaris*) bacteria. Lett. Appl. Microbiol..

[24] Chowdhuri AR, Tripathy S, Chandra S, Roy S, Sahu SK (2015). A ZnO decorated chitosan–graphene oxide nanocomposite shows significantly enhanced antimicrobial activity with ROS generation. RSC Adv..

[25] Dulta K, Koşarsoy Ağçeli G, Chauhan P, Jasrotia R, Chauhan P (2022). Ecofriendly synthesis of zinc oxide nanoparticles by carica papaya leaf extract and their applications. J. Clust. Sci..

[26] Yang L, Watts DJ (2005). Particle surface characteristics may play an important role in phytotoxicity of alumina nanoparticles. Toxicol. Lett..

[27] Ovais M (2018). Role of plant phytochemicals and microbial enzymes in biosynthesis of metallic nanoparticles. Appl. Microbiol. Biotechnol..

[28] Dong C (2016). Synthesis of stearic acid-stabilized silver nanoparticles in aqueous solution. Adv. Powder Technol..

[29] da Nunes ES (2014). Characterization of tetraethylene glycol passivated iron nanoparticles. Appl. Surf. Sci..

[30] Detsri E, Seeharaj P (2017). Colorimetric detection of glutathione based on phthalic acid assisted synthesis of silver nanoparticles. Colloids Surf. A.

[31] Velusamy P (2016). Synthesis of oleic acid coated iron oxide nanoparticles and its role in anti-biofilm activity against clinical isolates of bacterial pathogens. J. Taiwan Inst. Chem. Eng..

[32] Sawisai R, Wanchanthuek R, Radchatawedchakoon W, Sakee U (2019). Simple continuous flow synthesis of linoleic and palmitic acid-coated magnetite nanoparticles. Surf. Interfaces.

[33] Barman G, Maiti S, Laha JK (2013). Bio-fabrication of gold nanoparticles using aqueous extract of red tomato and its use as a colorimetric sensor. Nanoscale Res. Lett..

[34] Singh P, Kim Y-J, Zhang D, Yang D-C (2016). Biological synthesis of nanoparticles from plants and microorganisms. Trends Biotechnol..

[35] Manimaran M, Kannabiran K (2017). Actinomycetes-mediated biogenic synthesis of metal and metal oxide nanoparticles: Progress and challenges. Lett. Appl. Microbiol..

[36] Vanlalveni C (2021). Green synthesis of silver nanoparticles using plant extracts and their antimicrobial activities: A review of recent literature. RSC Adv..

[37] Rana A, Yadav K, Jagadevan S (2020). A comprehensive review on green synthesis of nature-inspired metal nanoparticles: Mechanism, application and toxicity. J. Clean. Prod..

[38] Singh A (2020). Green synthesis of metallic nanoparticles as effective alternatives to treat antibiotics resistant bacterial infections: A review. Biotechnol. Rep..

[39] Gupta KK, Aneja KR, Rana D (2016). Current status of cow dung as a bioresource for sustainable development. Bioresour. Bioprocess..

[40] Sedefoglu N, Zalaoglu Y, Bozok F (2022). Green synthesized ZnO nanoparticles using *Ganoderma lucidum*: Characterization and in vitro nanofertilizer effects. J. Alloys Compd..

[41] Sutradhar P, Saha M (2016). Green synthesis of zinc oxide nanoparticles using tomato (*Lycopersicon esculentum*) extract and its photovoltaic application. J. Exp. Nanosci..

[42] Hashemi S, Asrar Z, Pourseyedi S, Nadernejad N (2016). Green synthesis of ZnO nanoparticles by olive (*Olea europaea*). IET Nanobiotechnol..

[43] Mohammadi FM, Ghasemi N (2018). Influence of temperature and concentration on biosynthesis and characterization of zinc oxide nanoparticles using cherry extract. J. Nanostruct. Chem..

[44] Xu J (2021). A review of the green synthesis of ZnO nanoparticles using plant extracts and their prospects for application in antibacterial textiles. J. Eng. Fibers Fabr..

[45] Nagarajan S, Arumugam Kuppusamy K (2013). Extracellular synthesis of zinc oxide nanoparticle using seaweeds of gulf of Mannar, India. J. Nanobiotechnol..

[46] Al Awadh AA (2022). Sustainable synthesis and characterization of zinc oxide nanoparticles using *Raphanus sativus* extract and its biomedical applications. Curr. Comput.-Aided Drug Des..

[47] Rahayu E, Wonoputri V, Samadhi T (2020). Plant Extract-Assisted Biosynthesis of Zinc Oxide Nanoparticles and Their Antibacterial Application.

[48] Jayarambabu N, Kumari BS, Rao KV, Prabhu Y (2015). Beneficial role of zinc oxide nanoparticles on green crop production. Int. J. Multidiscip. Adv. Res. Trends.

[49] Selim YA, Azb MA, Ragab I, Abd El-Azim HM (2020). Green synthesis of zinc oxide nanoparticles using aqueous extract of Deverra tortuosa and their cytotoxic activities. Scientific Reports.

[50] Gupta M, Tomar RS, Kaushik S, Mishra RK, Sharma D (2018). Effective antimicrobial activity of green ZnO nano particles of *Catharanthus roseus*. Front. Microbiol..

[51] Shubha JP (2022). Facile green synthesis of semiconductive ZnO nanoparticles for photocatalytic degradation of dyes from the textile industry: A kinetic approach. J. King Saud Univ. Sci..

[52] Rajisha, K., Deepa, B., Pothan, L. & Thomas, S. Thermomechanical and spectroscopic characterization of natural fibre composites. In *Interface Engineering of Natural Fibre Composites for Maximum Performance*, 241–274 (2011).

[53] Maity, D., Chandrasekharan, P., Feng, S.-S. & Jun, D. *Synthesis and Studies of APTES Functionalized Magnetite Nanoparticles*, 94–97 (IEEE, 2010).

[54] Masud RA (2020). Preparation of novel chitosan/poly (ethylene glycol)/ZnO bionanocomposite for wound healing application: Effect of gentamicin loading. Materialia.

[55] Li X, Song R, Jiang Y, Wang C, Jiang D (2013). Surface modification of TiO2 nanoparticles and its effect on the properties of fluoropolymer/TiO2 nanocomposite coatings. Appl. Surf. Sci..

[56] Song J (2013). Synthesis of graphene oxide based CuO nanoparticles composite electrode for highly enhanced nonenzymatic glucose detection. ACS Appl. Mater. Interfaces..

[57] Majeed Khan MA, Kumar S, Ahamed M, Alrokayan SA, AlSalhi MS (2011). Structural and thermal studies of silver nanoparticles and electrical transport study of their thin films. Nanoscale Res. Lett..

[58] Suganya P, Rajamohan C, Mahalingam P (2018). Synthesis and surface modification of zinc nano rods using vermiwash of *Eudrilus eugeniae* and functionalization to seed germination of green gram *Vigna radiata*. Mater. Res. Express.

[59] Srivastav A (2021). Effect of ZnO nanoparticles on growth and biochemical responses of wheat and maize. Plants.

[60] Xun H (2017). Zinc oxide nanoparticle exposure triggers different gene expression patterns in maize shoots and roots. Environ. Pollut..

[61] Sharma D, Afzal S, Singh NK (2021). Nanopriming with phytosynthesized zinc oxide nanoparticles for promoting germination and starch metabolism in rice seeds. J. Biotechnol..

[62] DAS, B., Yonzone, R., Saha, S., Murmu, D. K. & Kundu, S. Comprehensive assessment of ZnO, P and TiO2 nanoparticles sustaining environment in response to seed germination, antioxidants activity, nutritional quality and yield of Spinach Beet (*Beta vulgaris* var. bengalensis) (2022).

[63] Elhaj Baddar Z, Unrine JM (2018). Functionalized-ZnO-nanoparticle seed treatments to enhance growth and Zn content of wheat (*Triticum aestivum*) seedlings. J. Agric. Food Chem..

[64] Naseeruddin R (2018). Unprecedented synergistic effects of nanoscale nutrients on growth, productivity of sweet sorghum [*Sorghum bicolor* (L.) Moench], and nutrient biofortification. J. Agric. Food Chem..

[65] Prerna (2020). Morphological and optical characterization of sol-gel synthesized Ni-doped ZnO nanoparticles. Integr. Ferroelectr..

[66] Khodakovskaya MV (2011). Complex genetic, photothermal, and photoacoustic analysis of nanoparticle–plant interactions. Proc. Natl. Acad. Sci..

[67] Ma H, Williams PL, Diamond SA (2013). Ecotoxicity of manufactured ZnO nanoparticles—A review. Environ. Pollut..

[68] Wu Y (2020). Response of the intertidal microbial community structure and metabolic profiles to zinc oxide nanoparticle exposure. Int. J. Environ. Res. Public Health.

[69] Sinha R, Karan R, Sinha A, Khare S (2015). Interaction and nanotoxic effect of ZnO and Ag nanoparticles against ESBL and Amp-C producing gram negative isolates from superficial wound infections. Int. J. Curr. Microbiol. Appl. Sci..

[70] Zhang L, Jiang Y, Ding Y, Povey M, York D (2007). Investigation into the antibacterial behaviour of suspensions of ZnO nanoparticles (ZnO nanofluids). J. Nanopart. Res..

[71] Yu K-N (2013). Zinc oxide nanoparticle induced autophagic cell death and mitochondrial damage via reactive oxygen species generation. Toxicol. In Vitro.

[72] Mohd Yusof H, Abdul Rahman N, Mohamad R, Hasanah Zaidan U, Samsudin AA (2021). Antibacterial potential of biosynthesized zinc oxide nanoparticles against poultry-associated foodborne pathogens: An in vitro study. Animals.

