# Supplementary material for: Cow dung extract mediated green synthesis of zinc oxide nanoparticles for agricultural applications
Source: Sci Rep. 2022 Nov 27;12:20371. doi: 10.1038/s41598-022-22099-y (PMC9701797; doi:10.1038/s41598-022-22099-y)
Supplement: Supplementary file 1 — Supplementary Information. [file 41598_2022_22099_MOESM1_ESM.docx]

**Supplementary Materials**


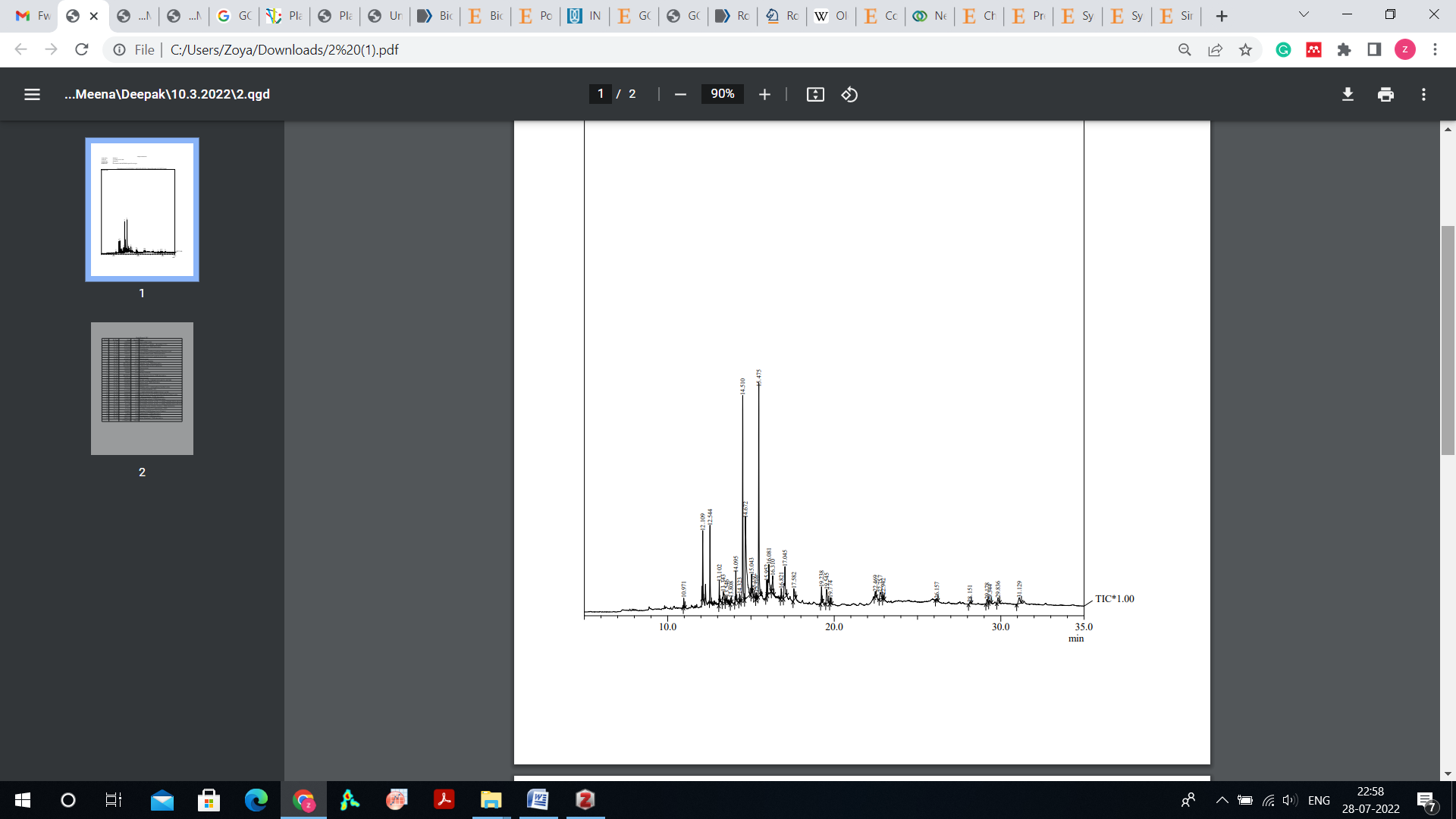
 **Fig 1-** Chromatogram of the cow dung extract

**Table 1-** List of compound present in the cow dung extract


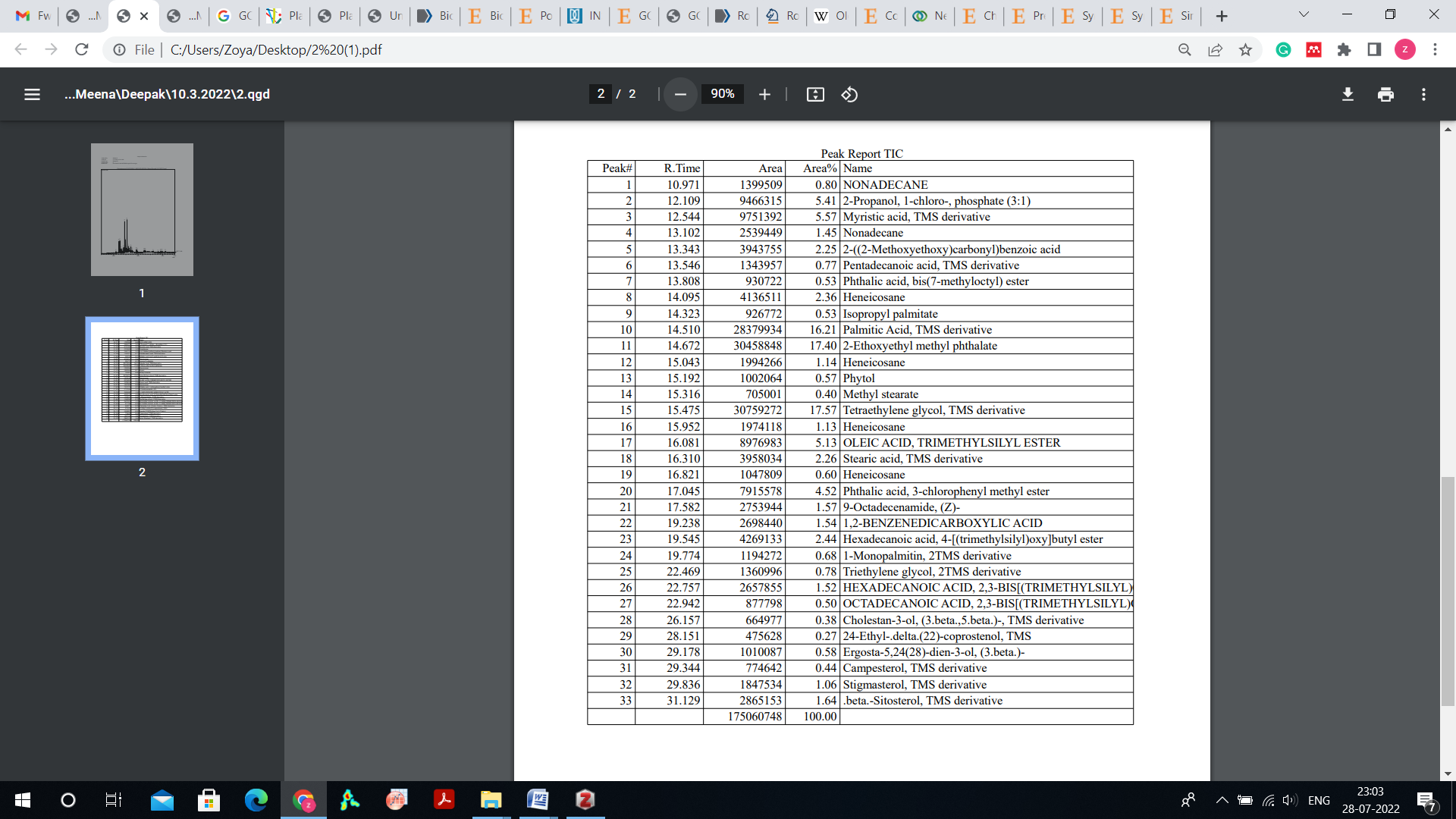


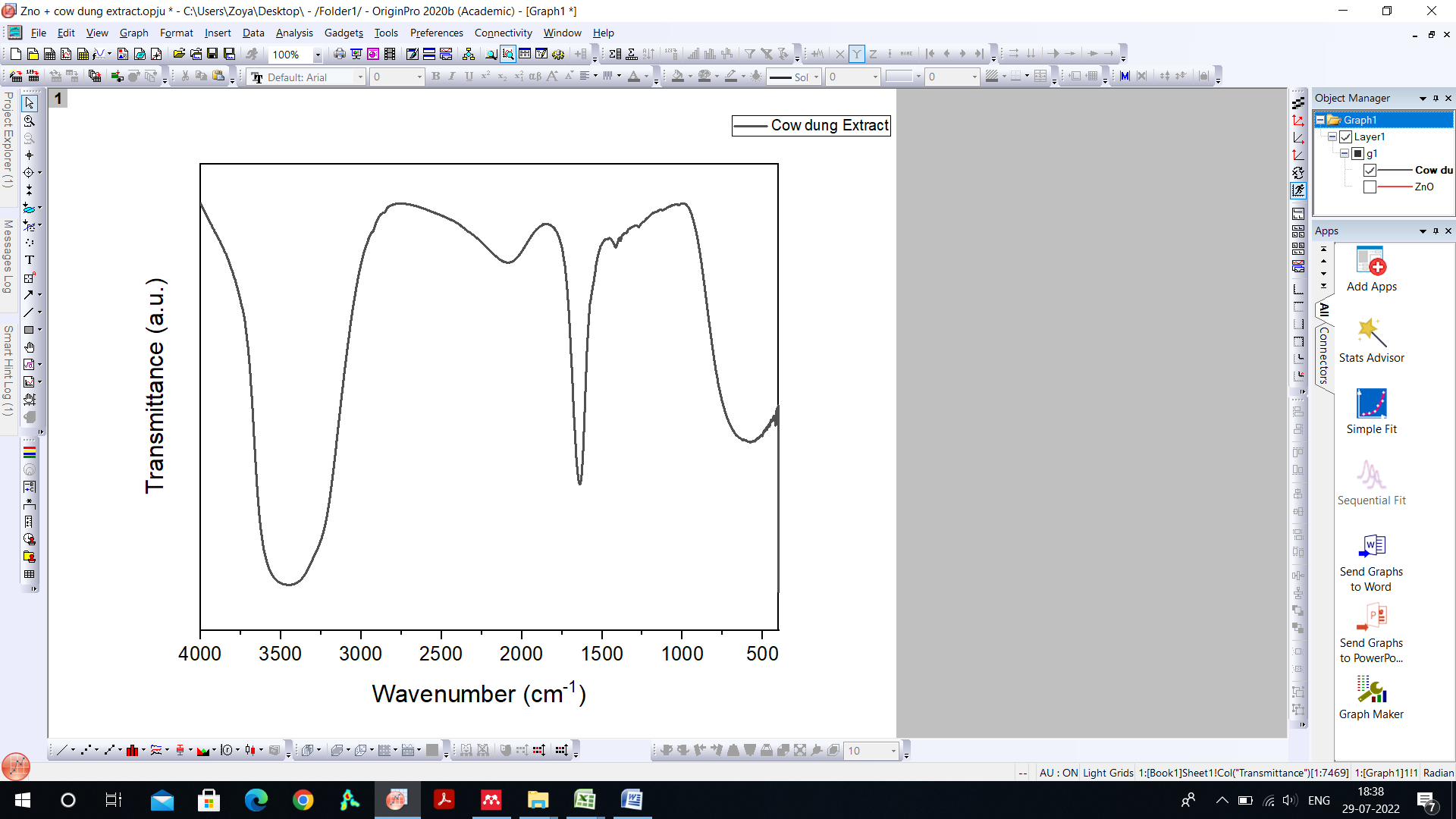


Fig 2- FTIR of Cow dung extract

**Figure 3** -UV-Visible spectrum of synthesised ZnO NPs at different pH. The sharp peaks were found at pH 8.

**Figure 4** -UV-Visible spectrum of synthesised ZnO NPs at different Temperature. The sharp peaks were found at temperature 80 ℃.
